# Genome Sequences of Mycobacteriophages Ebony and Holeinone

**DOI:** 10.1128/MRA.00186-19

**Published:** 2019-05-30

**Authors:** Jennifer Cook-Easterwood, Hailey Gase, Chioma Ngene, Joanna Katsanos

**Affiliations:** a Department of Biology, Queens University of Charlotte, Charlotte, North Carolina, USA; b Department of Chemistry, Queens University of Charlotte, Charlotte, North Carolina, USA; Queens College

## Abstract

Ebony and Holeinone represent the first mycobacteriophages isolated from Charlotte, NC, soil samples, using the host Mycobacterium smegmatis strain mc^2^155. Ebony has a 52,152-bp genome and showed similarity to other phages in the A11 subcluster. Holeinone has a 67,044-bp genome and showed similarity to other phages in the B2 subcluster.

## Abstract (precis)

Ebony and Holeinone represent the first mycobacteriophages isolated from Charlotte, NC, soil samples, using the host Mycobacterium smegmatis strain mc^2^155. Ebony has a 52,152-bp genome and showed similarity to other phages in the A11 subcluster.

## ANNOUNCEMENT

Ebony and Holeinone (Hole In One) were isolated from different soil samples (collected at 35.247° N, 80.931° W and 35.178° N, 80.834° W, respectively) using the bacterial host Mycobacterium smegmatis mc^2^155 as part of the Science Education Alliance-Phage Hunters Advancing Genomic and Evolutionary Science (SEA-PHAGES) program (1). Mycobacterium smegmatis mc^2^155 was provided by the Bacteriophage Institute at University of Pittsburgh and was grown in 7H9 complete liquid medium at 37°C for 5 days. Phages were isolated using enriched isolation and then purified and amplified following the protocols provided in the Howard Hughes Medical Institute (HHMI) SEA-PHAGES Discovery Guide (https://seaphagesphagediscoveryguide.helpdocsonline.com/home). Genomic DNA was isolated using a Promega Wizard kit, prepared for sequencing with an NEB Ultra II DNA kit, and run on an MiSeq instrument, yielding at least 1.1 million 150-base single-end reads per genome. Raw reads were assembled with Newbler version 2.7 (default settings) into a single contig for each phage and checked for completeness, accuracy, and genomic termini using Consed version 29 as previously described (2). Ebony had a genome length of 52,152 bp with a fold coverage of 3,187× and a G+C content of 63.8%, with 10-base single-stranded 3′ extensions (5′-CGGTCGGTTA-3′) (3). Holeinone had a circularly permuted genome with a length of 67,044 bp, a fold coverage of 2,992×, and a G+C content of 68.9%.

Both genomes were annotated using DNA Master (http://cobamide2.bio.pitt.edu/), and protein-coding genes were predicted using GLIMMER (4) and GeneMark (5), using the settings described in the HHMI SEA-PHAGES Bioinformatics Guide (https://seaphagesbioinformatics.helpdocsonline.com/home). Starterator (http://seaphages.org/software/) was used to predict gene starts, and protein function was determined using BLASTp (6) and Phamerator (7). ARAGORN (8) was used to analyze tRNA sequences. Ebony had 98 protein-coding regions, 1 tRNA, and 1 frameshift in the tail assembly chaperone genes. Holeinone had 90 protein-coding regions and no tRNA or frameshifts.

The bacteriophages Ebony and Holeinone represent the first mycobacteriophage species isolated from Charlotte, NC, soil samples. Ebony is part of the A11 subcluster. A Clustal Omega multiple alignment using default settings indicates there is a 98.10% average nucleotide sequence identity (ANI) between Ebony and Et2Brutus and a 97.24% average nucleotide identity (ANI) between Ebony and Mulciber. Holeinone is part of the B2 subcluster, and there is a 98.89% ANI with LizLemon and a 97.18% ANI with Godines.

### Data availability.

GenBank and SRA accession numbers are listed in Table 1.

**TABLE 1:** GenBank and SRA accession numbers and genome assembly results

Phage name	GenBank accession no.	SRA accession no.	Avg coverage (×)	Cluster	Genome length (bp)	G+C content (%)	No. of genes
Ebony	MH338236	SRX5352623	3,187	A11	52,152	63.8	98
Holeinone	MG812490	SRX5352622	2,992	B2	67,044	68.9	90
